# Syndromic Surveillance

**DOI:** 10.3201/eid1007.031035

**Published:** 2004-07

**Authors:** Zygmunt F. Dembek, Dennis G. Cochrane, Julie A. Pavlin

**Affiliations:** * Connecticut Department of Public Health, Hartford, Connecticut, USA;; † Morristown Memorial Hospital, Morristown, New Jersey, USA;; ‡ Walter Reed Army Institute of Research, Silver Spring, Maryland, USA

**Keywords:** syndromic, infectious disease, surveillance, bioterrorism, epidemiology, anthrax, smallpox, SARS, West Nile virus, hospital emergency department, letter

**To the Editor:** As public health practitioners directly involved in constructing, maintaining, and interpreting syndromic disease surveillance systems, we offer the following comments on the Buehler et al. article, "Syndromic Surveillance and Bioterrorism-related Epidemics" (1). In general, this article was well-crafted. It reviewed the potential for syndromic surveillance to detect various diseases of bioterrorism, specifically an anthrax event based on the inhalational anthrax cases of 2001. However, the reader may conclude that hospital-based syndromic surveillance is potentially ineffective and unproven.

Buehler et al. describe how, within 18 hours, a presumptive diagnosis of anthrax would prompt a full-scale response. We think that functional syndromic surveillance can respond to the rapid onset of hospital-based disease. To isolate and positively identify *Bacillus anthracis* from a blood culture would take ≈48 hours. Syndromic surveillance should detect a large number of cases within 24 hours. A fully functional hospital syndromic surveillance system that uses automated analysis (such as the daily emergency department–based surveillance with SaTScan in New York City) should identify a substantial increase in a relevant syndrome within 12 to 24 hours after data submission (2). A continued daily rise in any disease category would most certainly set off alarms in a syndromic surveillance network. If active statewide laboratory surveillance is included in syndromic surveillance, such as the gram-positive rod surveillance conducted in Connecticut (3), this surveillance should rapidly detect even single cases of anthrax concurrent with the presumptive diagnosis within the hospital.

The authors also state that syndromic surveillance would not detect outbreaks too small to trigger statistical alarms. The combination of active and passive surveillance in the hospital admissions–based syndromic surveillance in Connecticut allows a number of syndromes to be tracked immediately upon notification; these syndromes include pneumonia and acute respiratory disease in healthcare workers admitted to a hospital, all disease clusters, and fever with rash illness. This system is very flexible, and active surveillance of other syndromes can be quickly instituted as required. This active surveillance component has been proven useful. The first 2 of Connecticut's 17 confirmed human cases of West Nile virus during 2002 were discovered in August when a health director, who regularly monitored the syndromic admissions data for the hospital in his municipality, requested immediate West Nile virus testing from the hospital's infection-control department when he received two late summer reports of neurologic illness.

Buehler et al. state that specificity for distinguishing bioterrorism-related epidemics from more ordinary illness may be low because the early symptoms of bioterrorism-related illness overlap with those of many common infections. Illness specificity can be modulated within a syndromic surveillance system by making changes in the definition of the information requested, the method of analysis used, or by incorporating varying amounts of active surveillance into a passive reporting system. In Connecticut, annual rates of hospital admissions for pneumonia and respiratory illness have significantly increased (>3 standard deviations) during winter months. These increases have corresponded temporally with peaks in laboratory-confirmed influenza reports and in our state-based and the national sentinel physician influenzalike illness reports. Similarly, in the military-based syndromic surveillance system, respiratory outbreaks are detected by monitoring routine outpatient visits and pharmacy prescriptions. Absolute numbers of visits, as well as percentage of visits, to primary care clinics for influenzalike illness provide up-to-date information on respiratory disease conditions at military installations in both active-duty personnel and family members.

Connecticut has added additional active surveillance categories to its syndromic surveillance for potential SARS cases by gathering extensive data on all healthcare providers hospitalized with respiratory illness. In the absence of an identified pathogen, the entire United States was conducting syndromic surveillance for SARS during the spring of 2003.

What are existing alternatives to rapid, patient-based reporting through syndromic surveillance for bioterrorism and emerging illness? Will individual physicians (i.e., the "astute clinicians") truly recognize an increase of nonspecific symptoms among their patients in time to warn public health authorities of an impending bioterrorism event? During the past 4 years in the U.S. military population, unless disease was extremely severe with high rates of hospitalization, virtually no outbreaks of infectious diseases detected by syndromic surveillance were reported to public health officials, even when effective preventive measures existed. Our experience leads us to encourage states and municipalities to develop functional, patient-based syndromic surveillance systems and discover both their limitations and their possibilities.
