# Supplementary material for: Syndromic Surveillance
Source: Emerg Infect Dis. 2004 Jul;10(7):1333–4. doi: 10.3201/eid1007.031035 (PMC3323334; doi:10.3201/eid1007.031035)
Supplement: Related texts

# Reply: Syndromic Surveillance

**DOI:** 10.3201/eid1007.040125

**Authors:** James W. Buehler, Ruth L. Berkelman, David M. Hartley, Clarence J. Peters

**Affiliations:** * Emory University, Atlanta, Georgia, USA;; † University of Maryland School of Medicine, Baltimore, Maryland, USA;; ‡ University of Texas Medical Branch, Galveston, Texas, USA

**In reply:** The thoughtful letter of Drs. Dembek, Cochrane, and Pavlin draws attention to several key themes emerging in the ongoing dialogue about the utility and role of syndromic surveillance. First, as illustrated by their work, the growing body of experience in conducting syndromic surveillance should advance this dialogue beyond the hypothetical framework described in our manuscript to a more evidence-based assessment of epidemic detection. Second, in the absence of a bioterrorism-related illness since 2001, the utility of syndromic surveillance for detecting naturally occurring events is coming into greater focus, particularly for detecting the onset of anticipated seasonal upswings in infectious diseases, including West Nile virus disease, gastrointestinal illness, and influenza.

Syndromic surveillance coupled with follow-up investigations can assist clinicians by alerting them to communitywide problems likely to be manifest among their patients. This recognition may occur at the hospital level, as reported by Dembek et al. in the initial recognition of West Nile virus disease in Connecticut in 2002, or at the community level, as illustrated by public health alerts in New York City to notify clinicians about viral gastrointestinal illness (1). Multiple studies have documented that newer syndromic surveillance systems can recognize the onset of the annual influenza season (2), but it is not clear what these systems add to existing syndrome-based systems that track "influenzalike illness" as part of a larger array of influenza-specific surveillance methods. While Dembek et al. note that syndromic surveillance has detected multiple outbreaks that would have been otherwise unrecognized, Sichel et al. observed that syndromic surveillance did not detect outbreaks recognized through more traditional means (1). This discrepancy emphasizes the need to further assess the characteristics of epidemics and surveillance systems that favor detection by using syndromic methods.

We recommend distinguishing between the increasing practice, prompted by concerns about bioterrorism, of syndromic surveillance for epidemic detection and the longstanding and common practice of using syndrome-based case definitions in public health surveillance. Such case definitions have been used in situations in which a wide net is cast to identify potential cases of a particular disease (e.g., acute flaccid paralysis as part of global efforts to eradicate poliomyelitis [3], liver disease associated with a new therapy for latent tuberculosis infection [4], and inhalational anthrax in New Jersey in 2001, after bioterrorism-related cases were clinically detected [5]), when resource and infrastructure constraints do not allow routine use of laboratory-based definitions (e.g., surveillance for sexually transmitted diseases in infrastructure-weak countries [6]), and when surveillance is initiated for a new disease of unknown origin (e.g., toxic shock syndrome [7], AIDS [8], and severe acute respiratory syndrome [SARS] [9]). Although SARS surveillance did not represent syndromic surveillance according to this distinction, relationships between health departments and hospitals, fostered in establishing syndromic surveillance, likely facilitated SARS surveillance.

New guidelines offer an approach for evaluating syndromic surveillance systems, including what is learned from follow-up of statistical alarms and whether syndromic surveillance or other methods lead to the earliest detection of outbreaks (10). These guidelines also provide a framework for modeling exercises to test syndromic surveillance under various bioterrorism scenarios, supplementing experience gained from real-life, but typically less severe, seasonal illness or community epidemics. Eventually, this information should be useful in developing guidance for health departments seeking to determine whether and how to implement syndromic surveillance.

## References

[1] Sichel LS, Greenko J, Heffernan R, Weiss D. Field investigations of emergency department syndromic surveillance signals, New York City. Presentation at the 2003 National Syndromic Surveillance Conference [cited 2004 Jan]. Available from: http://www.syndromic.org/pdf/con2-LS-8b.pdf15717390

[2] Sosin DM. Syndromic surveillance: the case for skillful investment. Biosecur Bioterror. 2003;1:247–53. 10.1089/15387130377186144115040204

[3] Centers for Disease Control and Prevention. Global progress toward certifying polio eradication and laboratory containment of wild polioviruses, August 2002–August 2003. MMWR. 2003;52:1158–60.14647019

[4] Centers for Disease Control and Prevention. Public health dispatch: update: fatal and severe liver injuries associated with rifampin and pyrazinamide treatment for latent tuberculosis infection. MMWR Morb Mortal Wkly Rep. 2002;51:998–9.12455909

[5] Tan CG, Sandhu HS, Crawford DC, Redd SC, Beach MJ, Buehler JW, Surveillance for anthrax cases associated with contaminated letters, New Jersey, Delaware, and Pennsylvania, 2001. Emerg Infect Dis. 2002;8:1073–7.1239691810.3201/eid0810.020322PMC2730289

[6] UNAIDS/WHO Working Group on Global HIV/AIDS/STI Surveillance. Guidelines for sexually transmitted infections surveillance [monograph on the Internet]. 1999 [cited 2004 Jan]. Available from: http://www.who.int/emc-documents/STIs/docs/whocdscsredc993.pdf

[7] Osterholm MT, Forfang JC. Toxic-shock syndrome in Minnesota: results of an active-passive surveillance system. J Infect Dis. 1982;145:458–64. 10.1093/infdis/145.4.4587069226

[8] Centers for Disease Control and Prevention. Current trends update on acquired immune deficiency syndrome (AIDS)—United States. MMWR Morb Mortal Wkly Rep. 1982;31:507–8, 513–4.6815471

[9] Centers for Disease Control and Prevention. Outbreak of severe acute respiratory syndrome—worldwide, 2003. MMWR Morb Mortal Wkly Rep. 2003;53:226–8.12665115

[10] Centers for Disease Control and Prevention. Framework for evaluating public health surveillance systems for early detection of outbreaks; recommendations from the CDC working group. MMWR. 2004;53(No. RR-5):1–11.15129191
